# Intimacy, Anonymity, and “Care with Nothing in the Way” on an Abortion Hotline

**DOI:** 10.1007/s11013-022-09810-4

**Published:** 2022-11-28

**Authors:** Jennifer Karlin, Caroline C. Hodge

**Affiliations:** 1 Department of Family and Community Medicine, University of California, 4860 Y Street, Suite 2320, DavisSacramento, CA 95817 USA; 2 University of California, San Francisco School of Medicine, Department of Anthropology, University of Pennsylvania, 3260 South Street, Philadelphia, PA 19104 USA

**Keywords:** Medication abortion, Patient-centered care, Medical expertise, Patient-doctor relationship, Self-managed abortion

## Abstract

This essay is an ethnographic account of a volunteer, anonymous hotline of physicians and advanced practice providers who offer medical advice and guidance to those who are taking medications on their own to end their pregnancies. Attending to the phenomenology of caring on the Hotline reveals a new form of medical expertise at play, which we call “care with nothing in the way.” By operating outside the State’s scrutiny of abortion provision, the Hotline offers its volunteers a way to practice abortion care that aligns with their professional and political commitments and that distances them from the direct harm they see caused by the political, financial, and bureaucratic constraints of their clinical work. By delineating the structure of this new regime of care, these providers call into question the notion of the “good doctor.” They radically re-frame widely shared assumptions about the tenets of the ideal patient–doctor relationship and engender a new form of intimacy–one based, ironically, out of anonymity and not the familiarity that is often idealized in the caregiving relationship. We suggest the implications of “care with nothing in the way” are urgent, not only in the context of increasing hostility to abortion rights, but also for a culture of medicine plagued by physician burnout.

## Introduction

A nurse practitioner in the Midwest looks at her phone, opening an encrypted messaging app. It is 8:35 pm in January, and it is snowing outside. She just finished putting her kids to sleep and looks down at her phone to see if any messages have come in since she last checked 30 min prior.

Anonymous Incoming text (AIT): Hello

Standard reply (SR): Thank you for calling the M&A Line. If you have called between 8am to 11pm, we should be able to return your call within an hour. You can also text us.”

AIT: Okay. I have a major concern

The anonymous medical advisor (AMA) quickly replies in her app: “What is your concern?” She has an hour to get back to people when she is on call and has been working on the Hotline since 2 pm.

AIT: I took my misoprostol pill today and it didn’t even stay in for the whole 30 minutes on the side of my cheek to dissolve in my mouth

AMA: How long did it stay in your mouth?

AIT: Like a good 10 to 15 minutes

AMA: Then what happened? Did it dissolve or did you vomit? Or something else?

AIT: Dissolve

AMA: Dissolving is great. That’s what it’s supposed to do. That means it’s absorbed.

She waits for a few more minutes but does not get any additional texts. The practitioner thinks to herself: *I wonder where she is calling from? I wonder if it is snowing there and if she is calling us because she doesn’t want to go outside in the snow to a clinic tomorrow*? *It’s a good thing these medications are so safe and effective.*

In the next moment, she gets another text indicating that someone is calling. She calls them back and has a brief conversation and then writes into a separate encrypted data chat to all her other volunteer colleagues:

“Reason for call: took miso(prostol) yesterday morning, had heavy bleeding and cramping, but just light bleeding now.

Location of caller: 516

M or A: A

GA:

Age:

Upreg when:

If pills, source:

Other info: has appt scheduled for IUD insertion

Plan: reassured everything sounds normal”^1^

A few minutes later, she sees a note from another Hotline volunteer offering her some advice: “Hi! In case they aren’t going to the place that gave them the pills, they will do a urine pregnancy test before the IUD insertion and it will be positive, so you might want to warn them about that issue.” AMA responds “yup we talked about that!” Then, a moment later, the AIT texts again.

AIT @ 11:57pm: Yes, I am currently bleeding now, so it worked. Thank you!

AMA: Great.

AIT: But it’s like I feel good and bad. I am happy that the pills are working becuase it is not the right time for me to have a baby. I am not in the right relationship right now and it would be dangerous to bring the baby into the world right now. But, I feel bad because I want a baby eventually. Maybe I made the wrong decision?

AMA: It's common to have mixed emotions. A good resource is https://exhaleprovoice.org. They have people who are on the line that can listen to you and provide more emotional support if you need it.

AIT: Thank you!

It is a typical shift staffing the Miscarriage + Abortion Hotline (the M + A Hotline, or just the Hotline), a service provided by a volunteer group of anonymous, dedicated clinicians for people who are self-managing their miscarriage or abortion. While Hotline advisors draw on their medical knowledge and experience, the care delivered via the Hotline does not formally constitute the practice of medicine: providers do not provide prescriptions or abortion pills and they do not even refer directly to a site where pills can be obtained. They do not bill for services (or accept payment of any kind), nor do they operate in ways that would constitute a provider–patient relationship.^2^ Instead, the Hotline workers consider themselves akin to volunteer, advice-driven operations. Individuals contact the Hotline with a range of questions, from where to get abortion pills (callers are referred to a site that collates and vets sources for online pills called Plan C at https://www.plancpills.org/); to clarification about how to take the pills, whether their bleeding is normal (too much or too little), if their termination is complete, what to do about pain, or when to take another pregnancy test; how to handle complex or overwhelming emotions; or how to find care referrals in low-resourced settings. When an answer requires expertise outside the clinician’s remit, for instance, legal advice or mental health intervention, the callers are referred to the appropriate resources. Sometimes the interactions are managed in one text exchange; others require follow-up hours, days, or weeks later. Regardless, the clinician is meant to follow the individual caller’s lead, working to provide the best medical advice without compromising the callers’ anonymity, dignity, or wishes.

The Hotline, established in 2019, forms a part of the ever-shifting landscape of abortion care in the United States. Its founders, two primary care doctors, envisioned it both as a response to the acute crisis of recent aggressive abortion restrictions (Nash and Dreweke 2019) and as a project of reproductive justice, to redress the on-going historical crisis of racialized and class-based violence in medicine, generally, and reproductive healthcare, specifically (Cooper Owens 2017; Roberts 1997; Ross and Solinger 2017; Luna and Luker 2013). To the former point, more abortion restrictions (108) were enacted in 2021 than in any other year in history, including bans on abortion that flagrantly flouted the standard for legal abortion set forth in *Roe vs. Wade* in 1973 (a standard overturned when the Supreme Court decided *Dobbs vs. Jackson Women’s Health Organization* in the summer of 2022). To the latter point, the Hotline was envisioned as a high-quality alternative for those who have been historically discriminated against during in-person care, including individuals who identify as black and indigenous people of color (BIPOC), transgender and non-binary, and those with disabilities. As schemes of reproductive governance (Morgan and Roberts 2012) are increasingly punitive for pregnant and potentially pregnant people (Goodwin 2020), not to mention for the physicians who would care for them, a growing number of people have moved outside the medical sphere to self-source and manage their abortions (Ralph et al. 2020; Raifman, et al., 2021; Gill, et al., 2021).^3^ While individuals have always found ways to manage abortions on their own (Murphy 2012; Tone 2012; Solinger 2007), the existence of safe and effective medication abortion (commonly, a combination of mifepristone and misoprostol or misoprostol-only which can be sourced legally in some states, and readily on the internet) has led more and more experts to advocate for self-sourced and managed abortions as an important modality of abortion care (Donovan 2018; Moseson, et al., 2020; Karlin et al. 2021; Texas Policy Evaluation Project 2015). The M + A Hotline sits at this nexus of self-managed care and medical expertise, leveraging providers’ deep medical experience with abortion to support and reassure people whose abortions are happening beyond the confines of the clinic. Since its founding, utilization of the Hotline has increased steadily every month; they currently estimate they have assisted over 1200 people (Baker 2022).

In this essay, we consider how Hotline providers operate within a different framework of care and expertise than they do when medical care is delivered in the clinic. We argue that the Hotline incorporates aspects of medical expertise but does not function as an institutional space like the clinic, where many of the Hotline volunteers work for their formal employment. Instead, the Hotline offers its volunteers a way to provide care that aligns with their political and professional commitments to reproductive justice, offers a means of more closely achieving their idealized notions of the “good doctor,” and provides a place to provide abortion care without being complicit in state violence. Understanding the mechanics of the Hotline and its volunteer participants creates a means for considering more broadly how the enmeshment of the state into clinical practice functions to the detriment of both patients and clinicians. Our analysis follows the providers’ experiences of and reflections on caring on the Hotline, where their constructions of what good care comprises both shed light on the narrow question of the increasingly untenable conditions of providing abortion care in the United States and demonstrate how providers relate to formative structures and agency in a broader sense.

The arguments we advance in this paper are based on eighteen months of observations of interactions in the “floating space of electronic exchange” (Redfield 2013) of the Hotline (both between providers and callers and among the providers themselves), the structured protocols developed by the providers who staff the Hotline, and a series of interview questions sent via email to the providers about their experiences on the hotline, a quarter of whom replied. We directly observed callers’ interactions with Hotline providers over digital media, but we were not physically present with providers as they took calls. We have not maintained records of these interactions nor do we have access to the personal information of the volunteers or callers. We have included contextual details in the vignettes (like where the providers are located and what they are doing as they answer Hotline calls) to enliven a phenomenological sense of the social world of the Hotline (De Leon 2015). The clinicians who staff the Hotline are distributed around the country, and they pick up shifts intermittently as they go about their lives, making sustained in-person participant observation logistically challenging. In all, our ethnography is more likened to digital ethnography that leverages technological connection to trace the dynamics of social worlds online (Murthy 2011; Hjorth et al 2017) than it is to classic ethnographic fieldwork.

Importantly, this digital orientation has allowed us to do an ethnography of the Hotline without compromising the privacy of the providers. Many of the clinicians who staff the Hotline have not disclosed their participation on the Hotline to their wider personal and professional networks. Abortion care is so stigmatized in the United States—providers have regularly been the subject of harassment, occasionally spilling over into violence (Jefferis 2011; Russell-Kraft 2019; Doan 2007). Having an ethnographer tail these providers in person while they took shifts would compromise providers’ and callers’ privacy. Insofar as The Hotline’s paradigm places the privacy of the callers and volunteer providers as paramount, and seeks to be unobtrusive without compromising accuracy, the digital ethnographic methods we employ aim to mirror these logics of care (Mol 2008). Our methods avoid compromising anonymity of the callers and volunteers but more so, allow us to avoid participating, even obliquely, in discourses about abortion that mischaracterize and sensationalize the people who have them (National Abortion Federation 2003) or those who provide aid along the way.

A remarkably polarized facet of our political discourse, abortion is a familiar flashpoint in the so-called Culture Wars in the United States. Over the past four decades, social scientists have elucidated the cultural and political framings of abortion that propel it to such public prominence. They have excavated how anxiety about abortion serves as a proxy for unease with shifting gender roles (Luker 1984; Ginsburg 1989; Andaya and Mishtal 2016) and is entangled with eugenic impulses (Kline 2005; Kluchin 2011) or racial animus (Hogue and Langford 2020; Roberts 1997). Research on reproductive technologies has revealed the way technological innovation reworks social relations and normative expectations of gender and parenthood, like fetal ultrasound, for instance, which reorients popular discourses of fetal personhood (Petchesky 1987; Taylor 2008; Mitchell 2001; Morgan and Michaels 1999). The richest of these analyses trace out how policy choices, institutional actors, economic incentives, historical trajectories, and cultural forces coalesce into schemes of what Morgan and Roberts call “reproductive governance” (2012), which differentially curtail, control, and direct the reproductive lives of people along racialized and classed lines (i.e., producing “stratified reproduction,” (Colen 1995)). This research often foregrounds the experiences of patients, exploring their moral reasoning (Rapp 1999; Gammeltoft 2002; Gilligan 1993[1982]), experiences of stigma (Ellison 2003; Kumar et al. 2009), the barriers they face to accessing care (Singer 2020; Oaks 2003; Lane et al. 1998), or the long-term material and emotional consequences of abortion (or the inability to access one) (Foster 2020; Biggs et al., 2017). If one were interested in understanding the care provided by the Hotline more generally, one would undoubtedly need to speak with people who have availed themselves of its services, or develop some means of tracking outcomes, clinical or otherwise. These data would surely contribute productively to our understanding of the contemporary landscape in which people in the US choose and access abortions (Kortsmit, et al., 2021; Jones et al. 2021; Conti and Cahill 2019; Moseson, et al., 2021). Our focus exclusively on providers’ experiences of the Hotline is both an ethical-methodological choice, as asking patients to participate in research would interfere with the Hotline’s ethos of supporting people self-managing abortion as unobtrusively as possible, and a conceptual one: we are interested in understanding how it feels to provide care in such a space and charting the shifting epistemological stance towards medical expertise that accompanies these phenomenological experiences.

We join a smaller literature attentive to providers’ experiences of abortion care, which traces, for instance, the emotional and moral impacts of providing abortion care (Martin, et al., 2017), the harassment and stigma faced by abortion providers (Harris, et al., 2011), and the deputization of providers into schemes of reproductive governance (Mercier et al. 2016; Buchbinder 2016; Singer 2016). Beyond the context of abortion, a rich literature probes the production of physician subjectivity in the clinic, examining how, for instance, the structure of medical education (Good and Jo, 1995; Davenport 2000; Holmes et al. 2011), or contexts of material scarcity (Wendland 2010; Livingston 2012), or ethical uncertainty (Roberts 2012; Rapp 1999), affect the way that physicians conceptualize their professional identities and positionality within the clinic. Our ethnography develops similar themes as it explores the mechanics of care and the conditions of possibility for “good doctoring,” although our analysis is located outside the formal clinic. The medical care on the Hotline is different in kind from clinical work not only because it occurs outside a brick-and-mortar clinic, but because it makes clear the violence done upon health care practitioners who can often no longer practice in alignment with their ethical commitments as healthcare providers within the formal clinical space. It shows us what care looks like when bureaucratic impediments become so burdensome that providers must find a way to strip them away.

The special issue in which this essay appears centers on the role of dual training anthropologists and physicians and how insider–outsider status shapes the ethnographies of and in the clinic. Here, our embodied experiences as a physician and a physician in-training are particularly useful as we have been professionalized ourselves into the models of “good doctoring,” and we have direct knowledge of how the bureaucratic frustrations of conventional care can lead to internal conflicts when aiming to provide quality, unencumbered care. For example, we have been compelled to answer in the paternalistic cliché “because that’s the way it is” when asked by patients why we are documenting unnecessary information about them in our charts, or why we are performing confirmatory tests even after they have reliably reported their symptoms or test results to us. As a result, our patients wonder why they are not trusted by us, and we feel that we have let them down. Moreover, we know the exhaustion from having to spend much of our clinical time doing behind-the-scenes “social work” which can occupy more time and lead to failures of “medical practice” due to the artificial boundary placed between the two domains (Karlin 2022). We have felt the frustration of slamming into the bureaucracy that organizes medical care, from institutional policy to insurance policies, to public policy when trying to care for our most precarious patients (ibid) especially when we cannot get necessary procedures, tests, and medications for our patients due to access limits. While experiencing these frustrations in our everyday work, we simultaneously identify the larger political and structural factors limiting care as anthropologists of medicine. Being in the role of ethnographer and caregiver helps us to recognize the limits of clinical care within institutional spaces and to appreciate the burdens of caregiving that are imparted on us through our training and working environments – burdens that are so much more exaggerated and stigmatized in abortion care—and we can empathize with the Hotline volunteers’ desire to find a way to be the “good doctors” to which they aspire. This joint phenomenological and epistemological stance of the physician-ethnographer is what makes this positionality and analysis compelling in this instance and in the accompanying articles in this issue.

## Beautiful and Heartbreaking: Care on the Hotline

On a Wednesday evening on the West Coast, a physician is eating dinner with her family when her encrypted application on her phone dings:

AIT: I had took misoprostol alone for an abortion and I bled within 30 minutes of taking the first 4 pills. I had blood and blood clots and the day after my pregnancy symptoms were gone and for the next 2 weeks as well, and now 3 weeks later almost 4, I feel nauseous and I took a pregnancy test on Monday and it came out positive. I have light bleeding.

AMA: Hello. Thank you for texting. Do you know how far along you were in your pregnancy when you took the misoprostol, or when your last menstrual period was?

AIT: I was 10 weeks pregnant then and would be 12 weeks now and will be 13 on Sunday. I have an appointment with my pediatrician next week but I am 17 years old and cannot get care on my own without telling my parents. But, I cant tell my parents. I don’t want my mom to find out.

AMA: I am not sure how you want to handle it with your mom and the appointment, but you should definitely be able to talk to the doctor alone and you definitely don’t need to say that you used the pills if you do not want to.

AIT: No, yea, I heard you can get into legal problems and stuff like that. I am going to put on the paper that I would like to talk to the doctor alone and tell him that I just had a miscarriage and I wouldn’t like my mom to know. If it [the pregnancy test] comes out positive even though I am not pregnant, will he still prescribe me my birth control?

AMA: It is hard to know what another doctor will do. He SHOULD still prescribe you birth control, but that doesn’t mean he will. He may see that you have a positive pregnancy test and be worried you are pregnant. If you tell him you had a miscarriage, he should understand that the pregnancy test could still be positive and prescribe birth control.

AIT: Thank you so much. I really needed somebody with medical experience to talk too. Google searches really scare you.

The doctor puts the phone down and turns to her partner:

“This is what I love about this care. Young people do not always know who to turn to and whom to trust. It is just so satisfying to help people get around the misinformation out there. I just had an adolescent call who fears talking to her own doctor because she doesn’t even know she is allowed to talk to him about her reproductive health without her parents involved! At least I can tell her about her rights to confidential care and that the doctor would have no way of knowing if she took pills to end her pregnancy or had a miscarriage without pills.”

Hotline volunteers take shifts in which they are responsible for answering callers’ questions. Individuals can choose whether they would like to call or text. The advisors never ask questions that they do not need the answer to (for instance, in the example above, the location of the caller was not asked because it was not necessary for providing the answers to the adolescent’s questions). Their replies aim to be compassionate, non-judgmental, and unassuming. To this end, the language that is used when interacting with callers is as vague as possible, attempting not to presume anything about the person calling until that information is provided by the caller themselves. Confidentiality is the watchword in these interactions: providers and patients are both anonymous, and callers are not asked anything that is not directly relevant to answering their questions. Addressing misinformation and ensuring callers know their rights are key to the information provided by Hotline volunteers.

The M + A Hotline is currently staffed by volunteer clinicians united by their commitment to providing abortion care. Some are primary care physicians or obstetrician gynecologists; some are nurse practitioners, nurse midwives, or physician assistants. Many have been providing medication and surgical abortions to patients for years. Some live and practice in the liberal coastal cities where abortion is relatively easier to access; others have built careers in parts of the country where abortion is the subject of intense legislative scrutiny. All are deeply attuned to the ways that increasingly onerous regulatory regimes not only alienate patients from the care they need, but also impinge on clinicians’ practice of medicine. The providers who volunteer do so because they are committed to the moral and political issue of abortion access. One provider explains, “I got involved in the Hotline because it was a needed resource. I stayed because, as restrictions have increased, it was a way to feel like I was a part of the solution for ensuring care for people in those restrictive states.” Some providers who volunteer to work the Hotline see it as an add-on to the abortion care they provide in other parts of their professional practice. For others, the Hotline is the only abortion care they can provide due to prohibitions in their local practice sites. Indeed, one provider explained that she started volunteering for the Hotline when she could no longer do abortion care in her home institution: “I care very passionately about a woman’s right to choose and about women having control over their reproduction in general. At the clinic I work at we stopped doing any kinds of abortions about five years ago because of threats to our federal funding, and that was very upsetting.” For her, the Hotline offers a way to continue honoring what she understands as a moral and political imperative to provide abortion care when the state’s regulations became too burdensome for her institution to shoulder.

Among the Hotline clinicians, this commitment to abortion access is animated by a larger sense of social and reproductive justice. As abortion rates are highest among those who are black and non-Hispanic and concentrated in those of lower income (Jones and Jerman 2017), the Hotline volunteers see their work as creating equity as many of the people calling the Hotline cannot afford or cannot access quality care due to their social circumstances. The Hotline offers a way to instantiate these values that are also common among abortion providers generally: “My career is focused on supporting people who are otherwise marginalized and going above and beyond to make them feel seen, normal, accepted, and respected. We really get to do that on the Hotline.” Another provider echoed this sentiment: “I was feeling very burnt out and wanted to recommit to the social justice mission I entered health care with…. Working on the Hotline is part of that social and reproductive justice agenda.” A third remarked:

“Honestly, most of what I do in medicine is reassurance—teaching people about resources that are out there to help them meet their goals and helping them think through choices and options. I help them to feel empowered in their bodies and their choices for what they consider healthy. That is what we are doing on the Hotline. It makes me feel like I can finally practice medicine in the way that I had hoped I could when I applied to medical school.”

For these providers, it is not just the technical provision of abortion care at stake: they are narrating a set of deep commitments that have shaped their whole careers in medicine. Staffing the Hotline offers a means of enacting these ideals, oftentimes when their clinical work does not.

The Hotline presents a practical and easy way for providers to live their commitments to providing abortion care. Because it operates outside the ideologically motivated regulatory regimes that govern abortion, it offers providers a much-needed way to escape the scrutiny of the state. While all medical care is subject to legal and regulatory oversight, abortion draws a disproportionate amount of political attention. Of course, a clinician’s location has profound material impacts on the shape of the abortion care that they can provide. Providers who practice in states like Kansas, for instance, are mandated by law to tell patients-seeking abortion that there is a link between abortion and an increased risk of breast cancer (even though this association has been rigorously disproven) and that personhood begins at conception (though of course, personhood is an issue of metaphysics rather than medical or legislative expertise) (Guttmacher Institute 2022). Even in more reliably abortion access states, clinical protocols organized around liability rather than what is scientifically necessary can be burdensome for providers to navigate. Many clinics in California, for example, have been wary to dispense mifepristone for a patient to take at home at their own timing and discretion and have forced clinicians watch patients swallow the mifepristone even though such supervision is unlikely to make any difference in safety or efficacy. Other clinics have required a transvaginal ultrasound to confirm the dating and location of the pregnancy, even though evidence confirms that this is unnecessary in most cases (Raymond et al., 2020). These requirements of in-clinic care are often due to clinics’ experience of being scrutinized rather than any implementation of the medical evidence. The legal regulatory and bureaucratic regimes that so tightly ensnare abortion providers in their clinical practice engender an affective terrain that is marked by threat, fear, and the sense of surveillance (Foucault 1995). Simply put, it often does not feel good to practice in this space, even if one believes in the work (Martin et al. 2017). One Hotline volunteer evoked this affective fog as she explained that the Hotline was valuable precisely because “it allows me to sidestep the *oppressive* state to help people in need” [emphasis added]. The Hotline emerges in these discussions as an otherwise (McTighe and Raschig 2019) that is continuous with providers’ expertise and with their orientation to care but configured in such a way that it does not reproduce the harms caused by over-regulation.

Part of what weighs so heavily on these providers is that by complying with state regulations and clinical protocols crafted from a defensive stance, they feel complicit in the violence that such regulations inflict. The Hotline does not suffer from this problem as it exists outside of the state bureaucracy. One provider noted while reflecting on the required, but medically unnecessary tests and procedures that shape in-person abortion care:

“In medication abortion care, many clinics have been making people swallow mifepristone (the first pill in a medication abortion) in front of them because they interpreted the REMS [Risk Evaluation and Mitigation Strategy] to read that the individual had to take the pill in the office, even though the REMS clearly said “dispense” in the office, not “administer.” These tasks are not for the benefit of the patient, are unnecessary, and I can see the harm being done. But, abortion care is so surveilled that everyone is ultra-careful with the rules. By forcing people to be directly observed swallowing a pill, I am essentially giving the message that they don’t know their bodies, that they can’t advocate for themselves, and that we don’t trust them to perform a basic task on their own.”

In her estimation, tasks that are performed not for the patient’s benefit, but for the provider’s institution (in terms of liability or profit, for instance) or the state, end up harming patients. The nature of the harm done to the patient by such regulations will vary: sometimes it might be acute and financial, other times it appears as the incremental assault on dignity, where a lifetime of interactions with a system not set up to serve racialized patients calcifies into distrust and alienation (Cooper Owens 2017). The providers experience this harm as they feel a sense of complicity and accompanying guilt; these negative emotions might be part of the internal conflict that leads to some of the burnout that is so prevalent in medicine today (Dzau et al. 2018 ). The agita aroused in these providers as they contemplate their deputization into the cruelty of the state has been documented by ethnographers in other contexts. Mara Buchbinder describes how abortion providers in North Carolina “script dissent,” creatively resisting the state’s ideological messaging when new mandatory counseling laws were enacted (2016). Carolyn Sufrin (2022), in this special issue and in her ethnography *Jail Care* (2017), powerfully narrates the ambivalence that marks the provision of medical care in jail, elucidating how choreographies of care both implicate the provider in the punitive role of the state and create space to refuse it.

For the Hotline providers, the affective terrain of abortion care – instantiated by the threat of surveillance, by an understanding of one’s complicity in state violence, by the bureaucratic headache of “jumping through hoops,” as one provider put it, and by a belief that all these factors are inhibiting one’s ability to be a good doctor – culminates in their feeling betrayed by the profession of medicine and its goals. The ways in which this regime of reproductive governance fails patients are well documented, but it also fails providers by knee capping their capacity to enact an ethico-politics of care (Rose 1999) grounded not only in their moral and political beliefs about abortion, but in their commitment to the Hippocratic Oath and the obligations to patients it compels. Another Hotline provider laments, “It’s sort of beautiful and heartbreaking to bear witness to so much suffering/obstacles to reproductive healthcare. It’s beautiful in the sense that I get to sort of commiserate with the patient and validate to them that it’s bullshit, but it’s also so heartbreaking.” It’s beautiful. It’s bullshit. It’s heartbreaking.

This kind of betrayal of physicians is, if not unique in contemporary medicine, then at least remarkable. It is far more common to identify loci where patients have been betrayed or failed by the medical system and the state; for instance, where political responses to disaster wildly fail to provide for those affected (Petryna 2002; Adams 2013) or where contested illness categories, like Chronic Lyme Disease (Dumes 2020), leave people suffering without access to basic treatment, let alone compassionate care. More generally, the loftiest ideals of medical practice often go unrealized in the everyday choreography of care, frustrating patients and their physicians alike. But, the problem is especially acute in the realm of abortion care in the US, as we see in the experiences of the Hotline providers, who feel let down by a system that makes it difficult, if not impossible, to practice in a way that aligns fully with their ideals of caregiving.

## Care with Nothing in the Way

Where bearing the surveillance of the state engenders an affective dissatisfaction with their medical work–derived both from the pressure of practicing under threat and the sense that such strictures inhibit their ability to care well for their patients–the Hotline emerges as a powerful foil to the clinic: it offers a space where the providers can live up to the ideals of a “good doctor,” even though (or perhaps because) it is not a clinical space. That is, Hotline volunteers say what makes the Hotline a site amenable to the provision of exceptionally good medical care is the fact that it exists outside the Kafkaesque regulatory regimes of the state.^4^ One provider makes this abundantly clear, explaining that she likes the Hotline because, “It is nice to get to give advice that is based entirely in scientific data and a patient’s own desire instead of based on state laws created without those things in mind.”

The fact that the Hotline offers a means by which providers can participate in abortion care beyond the scrutiny of the state does not mean the care is unstructured or unsupervised. The Hotline maintains its own operating protocol, collating the best-available evidence regarding medication abortions and miscarriage management and providing a heuristic for the delivery of care. Standardized responses to common questions make it easy for clinicians to quickly and efficiently address concerns and keep commonly needed resources close at hand. In addition to these practice guidelines, providers on the Hotline get real-time assistance from their peers. Because all the chats are visible to the whole network of providers, there is counterintuitively more provider oversight than in other kinds of patient care. Rather than impinging on the autonomy of providers, this real-time observation, and an on-going separate channel where providers can communicate, offers a model of collegial and collaborative teamwork. In this back chatter, providers not only share up-to-the-minute research and discuss how to handle certain situations to best support the caller, but also monitor one another’s language to make sure it remains unassuming and non-judgmental. For example, one of the repetitive reminders–especially for new volunteers–is to avoid the word “woman” when referring to the callers until they know the gender of the individual calling, as many of those who reach out to the hotline are transgender and/or non-binary individuals. Removing the cultural assumptions often embedded in institutional care models takes time and repetition.

Where the regulatory apparatus of the state can feel oppressive, providers feel the Hotline guidelines instantiate, rather than hamstring, good care. The protocols center the desires and needs of those that are asking for help; allow (even compel!) providers to follow the science; foreground education and the combatting of misinformation; and, perhaps most importantly, prioritize these values over profit (there is no profit!) or bureaucracy. To the first point, one provider observed: “In the clinic where I work, I have to spend time asking questions that I know will not improve my care of the individual in front of me. With the hotline, I can just ask questions that are pertinent to the care. We don’t ask people where they are calling from unless we need to know that to give them advice. We don’t ask them if they used birth control or not unless that would affect their specific outcome. We let patients lead the discussion.” This discussion is oriented around what the individual reaching out for help wants and is informed by a commitment to scientific evidence. For one provider, listening to the science motivated her to start working on the Hotline:

“I started working for the Hotline after participating in a study that provided a list of high-quality research articles showing the safety of self-sourced medication abortion.^5^ As a physician, my main priority is improving patient access to evidence-based patient centered care. Once doing a thorough look at the research on safety for self-sourced medication abortion, I realized that this was an important answer to maintaining patient access as the political landscape was increasingly restrictive. My time with the Hotline has further strengthened my resolve that this is a safe option for medication abortion for those who desire it.”

Her subsequent experience on the Hotline becomes its own kind of evidence: her experience staffing the Hotline corroborates what the research tells her.

One provider identifies the care she can provide on the Hotline as valuable because it is unyoked from profit motives: “I believe in direct-to-patient advice without having the arbitrary gatekeeping system of capitalist productivity attached to it.” This framing is central to the Hotline’s public image as well, with its website explaining that it is a team of experts “freely giving [their] time to you.” Preempting claims of profit motivation is especially important in abortion care, where a popular strategy of abortion opponents is to invoke accusations of “big abortion” (Cooper 2016). In all, the values which our interlocutors identify as being central to providing good care – listening to and centering the patient, following the science, and prioritizing those things above profit motives or bureaucratic incentives – are not specific to abortion care: rather, and importantly, they are widely cited hallmarks of high-quality medical care.

We have come to understand the care that providers are describing on the Hotline as “care with nothing in the way.” Specifically, this is care without the state, or even the clinic, in the way. In a Hotline interaction, everything is stripped away: there is only an individual-seeking advice, a care provider answering with empathy, and that provider’s expertise. The absence of the intrusion of the state is most salient to these providers, who appreciate the freedom of being able to exercise their expertise and to follow the science, without being compelled by the state, or their institutional bureaucracies, to act in ways that are directly at odds with their medical knowledge. Care on the Hotline is also free of the weight of overly technocratic biomedical care (Kleinman and Hanna 2008; Good and Jo 1995). There are no unnecessary ultrasounds, no compelled pregnancy tests, no questions that must be asked regardless of their clinical utility; only the provider and caller engaged in what Sufrin refers to as “the romanticized relational aspects of care” (2017, 22).

It is possible to read *care with nothing in the way* as gesturing to a kind of nostalgia for the “good ol’ days,” where the practice of medicine could be humane and empathic because it was undistracted by technology (Volpintesta 1992), and “human values” were primary (Naughton 1977). This nostalgia can seem pervasive: both authors encountered physicians in our training who bemoaned what they saw as the over-reliance on imaging and other diagnostic tests, over and against good history taking. And Joseph Herman (1998) observes that it is nothing new: physicians have always been reaching towards some idyllic past. Among the providers on the Hotline, however, what we see is not so much a longing look backwards (they are far too aware of the racialized, class-based, and gendered violence which both forms the bedrock of reproductive healthcare and continues to shape its practice today), but a sense of what good doctoring should be. The vision of care articulated by those on the Hotline is less a nostalgia than an affective orientation towards an ideal. That is, the Hotline, despite its distance from the clinic, is a place where the conditions of possibility for good doctoring can be met. In this way, the Hotline functions as a crucible for a kind of ethical self-fashioning (Shaw and Armin 2011). It is surely not the only such place in medicine, but the Hotline offers a particularly generative site for understanding what those conditions might be. Indeed, Carole Joffe (1995) reminds us that abortion care, writ large, is a domain where physicians have long understood their practices a matter of conscience (see also Harris 2012).

Unsurprisingly, doing good doctoring (or at least, feeling like the barriers to good doctoring are minimized) feels great. As one provider enthuses, “On the Hotline, I really feel like I get to be a beacon of light for these patients wading through the darkness of shame and lack of information. I also get to be incredibly kind and reassuring when the rest of the world is cruel and deceiving on these topics. I get moments of these in the clinic, but it’s with almost every call that I receive this feedback from patients.” The gratification providers can experience on the Hotline might explain why providers who are overworked and over-extended keep showing up there for free. Their payment is of a different kind—that of satisfaction of their moral obligations. Given that the scholarly literature on physician burnout commonly hypothesizes its causes to be logistical (e.g., over-long hours, the creep of administrative tasks, the burden of the electronic medical record) and its solutions to be individualized (e.g., mindfulness trainings, self-care programs) (West et al. 2018, 2016; Yates 2019), it is remarkable that the providers understand their work on the Hotline – additional labor for which they are uncompensated – as a means of ameliorating their burnout. In other words, the quality of work, not just the quantity of it, matters. Our interlocutors’ narration of betrayal and their discussion of burnout posit a cause of burnout that is more existential than logistical, due rather to a “lack of alignment… regarding values, mission, purpose and compensation” (Rothenberger 2017): namely, the mismatch these providers are experiencing between the kind of self they want to cultivate as provider, and the kind of self that their institutional position allows. The Hotline, by creating conditions in which providers can resolve the alienation they feel from their ideals of clinical practice by assuming additional work, thus, contributes an important nuance to the scholarship on physician burnout.

While *care with nothing in the way* taps into a broader mythology about what it means to be a good doctor, care on the Hotline remains distinct from other kinds of patient care in a few key ways. For one thing, touch – the therapeutic laying of hands – looms large in the imagination of the good doctor (Kelly et al. 2020; Bruhn 1978 ; Buchbinder 2022). The tactility of care is in part valorized as a means by which the patient and provider are drawn into relation with one another: touch is an intimate gesture. But care on the Hotline is touchless—there is no physical exam; provider and caller may even be separated by thousands of miles. The implications of touchlessness in *care with nothing in the way* are not only structural, but also epistemological. That is, on the Hotline, the only information available to the provider is that which the caller chooses to share: the practitioners cannot extract data on their own during an exam. Listening and attunement are essential, not just because it serves the patient, but also because it is one of the limited tools at the physician’s disposal.

The epistemology of diagnosis in biomedicine teaches providers to distinguish between “subjective” accounts of illness and “objective” ones. The medical gaze, as Foucault classically develops it (1989[1962], describes the epistemic formation of “objective” knowledge in medicine, joining a consideration of the production of scientific knowledge, to questions of physician subjectivation, to the institutions in which medicine is practiced. The “subjective” represents the patient’s narration of their illness, while “objective” tends to refer to the physician’s observations and the results of their physical exam or other tests (Holmes and Ponte 2011). In the model of care revealed by the Hotline, these genres of fact-finding collapse. The only data the provider can respond to are the patients’ narrations of their own embodied experience, which thus privileges a form of evidence too often discounted by physicians, especially when the patient is one with a uterus (Hoffmann and Tarzian 2001; Zhang, et al., 2021). Providers have only their previous experience and expertise (i.e., no clinical infrastructure), and their care of callers is both less concerned with “en-case-ing the patient” (Holmes and Ponte 2011) and more deeply attuned to the ways in which the medical gaze privileges a white, male perspectives. Even the “objective” signs that a provider would look for on exam must be communicated by the patient here. The primacy of listening in this model, which prioritizes the phenomenological—what the caller is experiencing—stands in stark contrast to the Foucauldian medical gaze, which relies on creating objects and codifying those objects as facts through a process that merges sight and utterances into a form of knowledge which gains its power from classification and allows the physician to make claims to objectivity (Daston and Galison 2010). This new arrangement of medical perception, a new episteme, along with the fundamentally different mode of power it creates, provokes the cultivation of a new kind of scientific or expert self. *Care with nothing in the way* is, thus, care from a fundamentally different posture.

*Care with nothing in the way* is different than other modalities of good care in that both parties are anonymous.^6^ To the providers on the Hotline, this anonymity is paramount in creating the conditions for the care delivered. A provider elaborates, “Due to the anonymity of the Hotline, people who call us are free to be honest and tell us anything. As a clinician that allows us to truly put the caller first and help them safely access care without worry about administrators, insurance companies or other roadblock that we encounter in our daily clinics. Volunteering on the Hotline feels one step closer to liberating abortion and reproductive freedom for our communities.” Anonymity is functioning here as technology of intimacy, drawing the caller and provider together.^7^ The first effect of anonymity is as a means of obviating the shame, stigma, or fear a caller might harbor regarding their experience: anonymity unlocks a capacity for disclosure that aids in the therapeutic encounter without compromising the caller. The stigma callers may feel about abortion, specifically, is layered atop a general cultural narrative that frames female bodies as dirty, polluting, or even inherently shameful (Martin 1987) and a contemporary political landscape that makes disclosure of abortion risky. Disclosure of bodily function can be embarrassing or uncomfortable for patients in clinic, but Hotline callers are anonymous. If touch is a means of fostering intimacy in the traditional clinical encounter (where intimacy is understood as affective connection, see Berlant 1998), this frank discussion of flesh serves an analogous function on the Hotline, as one advisor notes: “I think part of the instant intimacy…is because we go right into bodily stuff: bleeding and clots and tissue and pain and nausea—it’s all so very personal and represents that something desired (freedom from the pregnancy) is going as it should. It is intimate. And freeing.” The body is the ground for connection, and the immediate and candid discussion of its emissions and idiosyncrasies, enabled by anonymity, draws the provider and caller close: disclosure produces intimacy (Jamieson 1998). This mode of intimacy is particularly important in a context where the “compassionate intimacy of care” (Sufrin 2017:103), cannot derive from the structure of clinic routines (ibid), or the slow work of gradual attunement to another’s affective life (Govindrajan 2018).

As these two providers make clear, anonymity and the intimacy it produces are nothing short of liberatory. In the first case, anonymity as a technology of intimacy is freeing for the caller, structuring a relation to the medical gaze that is less objectifying. In the second case, it is both the provider who is liberated, able to provide care without “roadblocks,” outside the medical-industrial complex, as well as abortion itself, wrested from the clinic and returned to the community.^8^ Anonymity creates the conditions in which care cannot be sidetracked, where all that matters is what the caller needs: this is *care with nothing in the way.*

In a culture of medicine that holds patient-centered care—where “patients are known as persons in context of their own social worlds” (Epstein and Street 2011)—as the ideal of clinical practice, it might seem counterintuitive to assert that anonymity might be crucial, even liberatory, in certain contexts. That is, that some of the providers on the Hotline understand anonymity to be a key part of why care on the Hotline is particularly effective, why they can be particularly good doctors in this space, invites us to re-consider conventional wisdom about what makes a good doctor, and what structures an effective and caring patient-provider relationship.

Patient-centered care (PCC), often painted as in tension with evidence-based medicine (Bensing 2000; Weaver 2015), is widely understood as a model of “good doctoring,” valuable on its own terms, but also because it serves as a corrective to a model of care that is de-humanized and overly technical, which is too apt to “view the person of the patient as an irritating distraction” from the pathology that needs addressing (Miles 2009). Proponents of PCC insist that good doctoring and strong therapeutic relationships must center the patient as a person, which is achieved at least in part, by listening to and respecting them, and involving them in decision making (Barry and Edgman-Levitan 2012). Intimacy, vulnerability, and deep connection are the substrate upon which a meaningful and productive therapeutic relationship can be built (Gordon and Beresin 2008). What it means to value “the whole person,” or to know them “in the context of their own social worlds,” as Epstein and Street (2011) put it, is often operationalized in medical education and practice by focusing on communication skills—for instance, teaching medical students to ask about a patient’s livelihood, hobbies, or children in their history taking—that aim to capture the patient as a psychosocial whole and to facilitate rapport between provider and patient (King and Hoppe 2013).

There are undoubtedly many clinical scenarios where this model of in-person, patient-centered care is invaluable, even critical, both in terms of patient outcomes and for provider satisfaction (Hashim 2017). Within the realm of reproductive health for instance, the research shows that many abortion providers derive fulfillment and inspiration from the relationships they form in-person with the patients they serve; likewise, many abortion patients report drawing comfort from the tender care they receive in person (Joffe 1995, 2009). Indeed, even a Hotline provider deeply invested in the work the Hotline remarked that she prefers in-person care: “I find that although I love that the Hotline exists to help people navigate [self-managed medication abortion], it isn’t as fulfilling as my clinic time. I miss the continuity and the ability to have deeper connections.” It is this deeper, personal connection – here mapped onto a temporality that extends, perhaps indefinitely as continuity of the patient-provder relationship – that is the engine of dominant models of patient-centered care.

Care on the Hotline, as this provider is well-aware, is different: its quality, not to mention its general contours, cannot be predicated on personal connection, facilitated by time or touch. Rather, we argue that the anonymity of the Hotline functions as a technology *of and for* intimacy. What we are suggesting is that intimacy in the clinical encounter need not be facilitated by a deep and personal knowledge of the other, or by physical proximity and therapeutic touch: there are other configurations of care, of patient-provider relationships, that can nevertheless be resolutely patient-centered, allowing providers to enact ideals of good doctoring. Indeed, it is not just Hotline providers who feel this model of care is high quality. Although our sense of patient experience is limited to what callers organically express in their texts to the Hotline, this preliminary data would suggest that most patients are highly satisfied by *care with nothing in the way*. The number of patients who express gratitude and satisfaction to providers is remarkable, as one provider made clear: “I’ve started to put quotes from patients in my data responses for everyone else to see, because people are so explicit about how grateful they are for our advice.” Explaining how gratifying this was to her, how good it makes her feel she added, “I get moments like this in the clinic, but it’s with almost every call that I receive this feedback from callers.” One patient messaged the hotline: “Thank you for your kindness, reassurance, and all of this information. I greatly appreciate you and just want you to know that I’ll never forget the compassion you showed me on one of the toughest days of my life < 3.” Another highlighted the stakes of such care today: “I am so relieved to talk to you, I’m in Texas and can’t even raise my voice to ask you questions because I’m worried a neighbor will hear. You’re doing god’s work, god bless you.” In a recent news article about care in Oklahoma after the *Dobbs* decision, an anonymous person recounting their abortion experience wrote specifically about their experience accessing the Hotline: “Almost daily, I called the Miscarriage and Abortion Hotline, and they were so kind, helpful, and informative. They broke it down for me scientifically and went through it piece by piece: what my body’s doing, how it’s healing, when to worry, and when not to worry. It’s been a really comforting experience to know that all these abortion resources and hotlines are out there to help you if needed.” (Popsugar 2022). Further evincing the value of anonymity to patients is the fact that some portion of people who contact the Hotline have received care in clinic first and choose to reach out to the Hotline when they have questions or issues, rather than contacting the clinic where they received their initial abortion care. Admittedly, more investigation into how patients experience the Hotline would be necessary to substantiate how they relate to *care with nothing in the way*. However, this form of research would be difficult as it would necessarily have to adhere to the Hotline’s ideals and not request accounts of satisfaction and other data from callers, as this intrusion of research, and its associated tainted history of ethical breaches particularly for people of color and those more vulnerable like prisoners and native populations (Roberts 1997, 2011; Owens 2017; Reverby 2009, 2012; Gamble 1997) could compromise *care with nothing in the way*.

As a model of care, we have shown that *care with nothing in the way* prioritizes what patients want and need from an encounter (there are no administrative or research duties to fulfill). Moreover, it puts individuals asking for care in the driver’s seat. Since callers control the terms of the interaction with their narrations of their symptoms as the only available clinical data, the power relation between provider and patient is redrawn. In this model of care, the provider inhabits their expertise from a fundamentally different posture. *Care with nothing in the way* does not repudiate the values of patient-centered care; rather, it challenges the norms by which those values are enacted. The model of care on the M + A Hotline suggests that the kind of care implicitly rejected by the patient-centered care movement—care that is direct, targeted, clinically efficient, and totally anonymous—can, in the right set of circumstances, be a model for high-quality, patient-centered care.

Parsing the dynamics of patient-centered care in contexts that are not traditional, in-person clinics is important as those modalities of care become more prevalent and necessary. Both the explosion of telemedicine during the COVID-19 pandemic (Ramaswamy et al. 2020) and the crush of abortion bans and restrictions following the overturning of *Roe vs. Wade* that have made the remote provision of abortion care more urgent offer the opportunity to explore how good care can be fashioned beyond the clinic. The model of care on the M + A Hotline suggests that such options need not be inherently inferior to their in-person analogs. Indeed, we might ask how the principles of *care with nothing in the way* might be transported back into the clinic, and how this integration might broaden our conceptualization of how patient-centered care might be operationalized in-person.

## Caring In Uncertain Times

Resources like the M + A Hotline are likely only to become more important in the coming months and years. We are barreling towards a future where abortion is more highly regulated and stigmatized than at present, where access is less even than ever (and it is already very uneven), and where getting help may become more dangerous for patients. From the passage of SB8 in Texas (a six-week abortion ban that deputizes private citizens into its enforcement, placing bounties on the backs of people “aiding and abetting” abortions), to the decision in *Dobbs vs. Jackson Women’s Health Organization* overturning *Roe vs. Wade* and *Planned Parenthood vs. Casey*, abortion advocates are preparing for a pronounced escalation in hostility to legal abortion (In Our Own Voice 2021). Self-sourced medication abortion and reaching out to resources like the M + A Hotline, may become a primary way that people access abortion in restrictive states. The future of abortion care raises important questions for providers committed to this work. How will they respond as they are further weaponized in the Culture Wars? What kinds of care will they be able to provide, and for how long? These questions are not abstract: our colleagues in Texas and other restrictive states corroborate the reporting that journalists are doing to document the harassment and heightened scrutiny faced by already beleaguered patients and providers.

For the Hotline volunteers, these menacing uncertainties raise concrete questions about the sustainability of the Hotline. How can it be staffed most effectively to respond to increased call volumes? What needs to change, if anything, about the scope of questions they respond to? One lesson that the Hotline makes particularly poignant is that the bracing constraints of political discourse about abortion in the United States, joined to the machinations of the medical-industrial complex, need not overdetermine the mechanics of abortion care. The Hotline offers a model of the otherwise (McTighe and Raschig 2019): competent, high-quality care that is delivered from a fundamentally different posture. It offers a different way to inhabit and wield one’s expertise, to structure an interaction between patient and provider. How durable is this model of care? As conditions on the ground deteriorate, will the Hotline continue to exist as a caregiving space where the ideal of the good doctor becomes graspable (Kleinman 2006)? Or will this space buckle under the strain?

The future of the Hotline bears not only on the landscape of abortion care in the coming years, but also offers, as we have argued, a means of unsettling pervasive assumptions about what constitutes good doctoring. We trace how the Hotline enacts a model of care that privileges callers’ subjective experiences over and against other modes of medical perception and that exists outside the scrutiny of the state and the medical-industrial complex. This offers providers a different way to inhabit their expertise and to orient towards their ideals of clinical practice. Along these lines, we seek to open a more general discussion about the contemporary practice of biomedicine. Specifically, in demonstrating how the anonymity of the Hotline functions as a technology of and for intimacy between callers and providers, this essay has implications for understanding both the pressing problem of physician burnout and for the delivery of care that is authentically patient-centered. Ultimately, we ask how *care with nothing in the way* might be transported into other clinical contexts. Can this restructured relationship between patient and provider be transferred back into the clinic to operate in face-to-face interactions? In a world where all kinds of clinical practice are increasingly under strain, exploring models of care oriented towards liberation feels ever more urgent.

## Footnotes

1 The data chat template for recording calls vaguely resembles what might go into the note a clinician would write in a patient’s medical record to document the visit. The key difference is that these health care providers only note what is essential, and nothing extraneous, to answer the caller’s questions. The abbreviations decoded: “M or A” = miscarriage or abortion, “GA” = gestational age, “Age” = age in years of caller, “Upreg when” = date of positive urine pregnancy test, “If pills, source” = refers specifically to abortion pills, usually a combination of mifepristone and misoprostol, or misoprostol only.

2 With the understanding that the work done by Hotline volunteers does not constitute the practice of medicine, legally speaking, throughout this article we will talk about “care” or “medical care” performed within the confines of the Hotline. This theorization of care in the space of the Hotline draws on a rich seam of anthropological scholarship that demonstrates empirically the ways in which the “care” delivered or experienced in each therapeutic space may exceed, refute, or re-work legal or regulatory regimes which delineate the realm of acceptable medical practice (see, e.g., Stevenson 2014, Sufrin 2017, Wendland 2010, McKay 2017, Livingston 2012). When we talk about care in this essay, we are not making an assertion of the practice of medicine in a legal sense, but in an ethnographic one.

3 It can be argued that almost all medication abortions are already self-managed as individuals who take misoprostol (either alone or in combination with mifepristone) do so outside of the doctors’ office without clinical oversight, per standard medication abortion protocols.

4 This sentiment is a powerful rebuke to anti-abortion legislators who insist that abortion restrictions are necessary to protect the health and safety individuals seeking abortion care, in short, to ensure good medical care (see Annapragada 2017).

5 See Karlin et al. (2021) for the study referred to by this physician.

6 The role that anonymity plays in the context of a hotline immediately draws to mind Lisa Stevenson’s exposition of “anonymous care” in the Canadian Arctic (2014). She draws an important distinction between professional contexts where anonymity may be appropriate, even essential, and “anonymous care,” which is care without a specific object: “to care anonymously requires being able to care intransitively, to be able to say ‘I care,’ without specifying for whom” (83). Although it is worth considering the degree to which abortion care in the US might constitute a form of “anonymous care,” our discussion of anonymity on the Hotline here is concerned with anonymity in the mundane sense, i.e., when identifying details have been obscured.

7 Ethnographers have documented how anonymity can function in more insidious ways. Emma Backe, an anthropologist who staffs and studies rape crisis hotlines, documents how her hotline’s insistence on anonymity frustrates repeat callers (of whom there are many), who must re-narrate their trauma each time they reach out for support (2018).

8 This context is itself an interesting site for a consideration of the interdigitation of intimacy and care, as much contemporary scholarship on intimacy focuses on the commodification of intimacy and the dynamics of intimate labor under capitalism (see e.g., Boris and Parreñas 2010, Constable 2009).
